# Transient blocking of NK cell function with small molecule inhibitors for helper dependant adenoviral vector-mediated gene delivery

**DOI:** 10.1186/s13578-015-0023-0

**Published:** 2015-06-11

**Authors:** Manjunatha Ankathatti Munegowda, Jim Hu

**Affiliations:** Department of Physiology & Experimental Medicine, The Hospital for Sick Children, Peter Gilgan Centre for Research and Learning (PGCRL), 9th floor, 686 Bay Street, Toronto, ON M5G 0A4 Canada; University of Toronto, Toronto, ON Canada

**Keywords:** HD-Ad, NK cells, Macrophages, Janus kinase inhibitor, NF-κB inhibitor

## Abstract

One major challenge in gene therapy is the host immune responses against viral vectors. Previous studies indicate the involvement of NK cells in stunted gene expression in viral vector mediated gene therapy. To understand the problem of the immune responses, we have developed an *in-vitro* co-culture system with human NK cell line, macrophages and airway epithelial cells. We showed that small molecule blockers, CAPE and ruxolitinib, for NF-κB and JAK-STAT pathways, respectively, significantly inhibited cytokine secretion by macrophages. When NK cells are co-cultured with helper-dependent adenoviral (HD-Ad) vector activated macrophages, IFN-γ cytokine expression by NK cells increased significantly, which was inhibited effectively by ruxolitinib and CAPE, and there was an additive effect when both inhibitors were used. We demonstrated that NK cells activated by cytokines produced by HD-Ad-activated macrophages kill HD-Ad vector transduced bronchial epithelial cells. This cell killing activity was significantly reduced by CAPE and ruxolitinib. Combination of these two inhibitors had an additive effect on inhibiting NK cell mediate killing of gene transduced cells. Transient inhibition of NK cell response at its peak may enhance sustained gene expression. Our data suggest that combination of CAPE and ruxolitinib may help in protecting gene transduced airway epithelial cells to prolong transgene expression.

**Electronic supplementary material:**

The online version of this article (doi:10.1186/s13578-015-0023-0) contains supplementary material, which is available to authorized users.

## Background

Our ability to detect genetic deletions and mutations that cause human diseases has immensely improved due to tremendous progress in human genetics [1, 2]. However, the current major hurdle to realize the full potential of therapeutic benefits from advancements in genetics is the slow progress in translating our knowledge into clinical therapeutic applications. Conceptually, correction of a genetic mutation/deletion that causes a disease should allow us to treat/cure the disease at its cause, not its symptoms, thereby, revolutionizing the human medicine. But, technically, it is difficult to deliver genetic materials, efficiently and safely despite the hype generated in early 1990s with only few success stories till now [3–5]. One of the major challenges faced in early gene therapy trials was the lack of novel delivery vectors with a high efficiency and easy production in high quality. There has been tremendous research in improving vectors to efficiently express genes in target cells [6, 7]. Our group has developed an efficient helper-dependent adenoviral (HD-Ad) vector to express the human cystic fibrosis transmembrane conductance regulator (CFTR) gene with epithelial cell specific K18 promoter for cystic fibrosis gene therapy [8].

Another major challenge has been the host immune responses that eliminate the cells transduced by gene therapy vectors [9, 10]. In airway gene delivery with adenoviral (Ad) vectors, apart from the physical barriers posed by mucus layer and tight junctions, both innate and adaptive immune responses were found to be a major problem that makes the transgene expression transient [10]. The innate immune response is caused by the viral capsid proteins and the adaptive immune response is largely caused by the leaky expression of viral genes in addition to the capsid proteins. To reduce the adaptive immune response, a major improvement was made to Ad vectors by creating the HD-Ad vector in which all viral genes are deleted [11]. We and others have shown that HD-Ad vectors show long term transgene expression with reduced immune responses in mice [12, 13]. Because of the deletion of viral genes, this type of vector has very large gene carrying capacity (37 kb) and can be used to deliver large genes or multiple genes which cannot be handled by other commonly used vectors. We have further developed aeorsolization protocol to achieve highly efficient gene delivery to rabbit lungs using HD-Ad vectors [14]. We have recently modified our protocol and achieved highly efficient vector delivery to pig lungs [8].

Since HD-Ad vectors contain the same capsid proteins as the conventional Ad vectors, they are expected to inflict innate immune responses. It is known that immune cells such as, macrophages [15, 16] and NK cells [17, 18] are involved in destroying gene therapy vectors or eliminating the transduced epithelial cells. Because robust levels of transgene expression can be maintained for a long time in rodents [6, 7], but was not achieved in large animal models, we need to understand how these innate immune cells affecting gene expression. Our studies with pig gene delivery show that IFN-γ is induced upon viral vector delivery [8]. Since NK cells are the major producers of IFN-γ *in vivo* [19], they are likely a barrier to sustained gene expression in pig airway. Thus, NK cell-mediated killing of gene transduced cells might be a major problem unnoticed in past clinical studies.

To understand the problem of immune responses we have developed an *in-vitro* co-culture system with human NK cell line, macrophages and airway epithelial cells. NK cell line, NK-92 is a human Natural Killer cell line derived from rapidly progressive non-Hodgkin's lymphoma patient's peripheral blood mononuclear cells [20]. THP-1 cells are monocyte cells line grown in suspension, they become attached once they are differentiated to mature macrophages in presence Phorbol 12-myristate 13-acetate (PMA) [21]. BEAS-2b, a cell line established from normal human bronchial epithelial cells. We used human cell lines in the study because of the lack of pig cell lines and reagents specific to pig cells. Eventually, HD-Ad gene therapy has to be tested in clinical trials; our results with human cell lines will be useful in designing human applications. To block NK cell, macrophage and epithelial cell interaction, and NK cell mediated killing of gene transduced cells, we targeted NF-κB and Janus kinase/signal transducers and activators of transcription (JAK-STAT) pathways. These pathways are critical for producing proinflammatory cytokines (such as, interferons, IL-6, IL-12, IL-15, IFN-γ) [22, 23]. We used small molecule blockers ruxolitinib and CAPE to block NF-κb and Jak-Stat pathways, respectively. Among the NF-kB inhibitors, CAPE [24] and Bay 11–7082 [25] are good candidates because of their potency. We used CAPE because Bay 11–7082 can only be dissolved in DMSO, because DMSO alone is shown to have influence on cell growth [26]. There are a quite number of inhibitors available for Jak-Stat pathways. We used Ruxolitinib which is a very potent inhibitor for Jak1 and Jak2 [27] and it is currently used in clinics for human therapy for myeloproliferative neoplasms [28–30]. In this paper, we demonstrated that these small molecule inhibitors can effectively block the activation of NK cells by HD-Ad vectors in our co-culture system.

## Results

### Ruxolitinib and CAPE block activation of macrophages by HD-Ad vectors

**Fig. 1:**
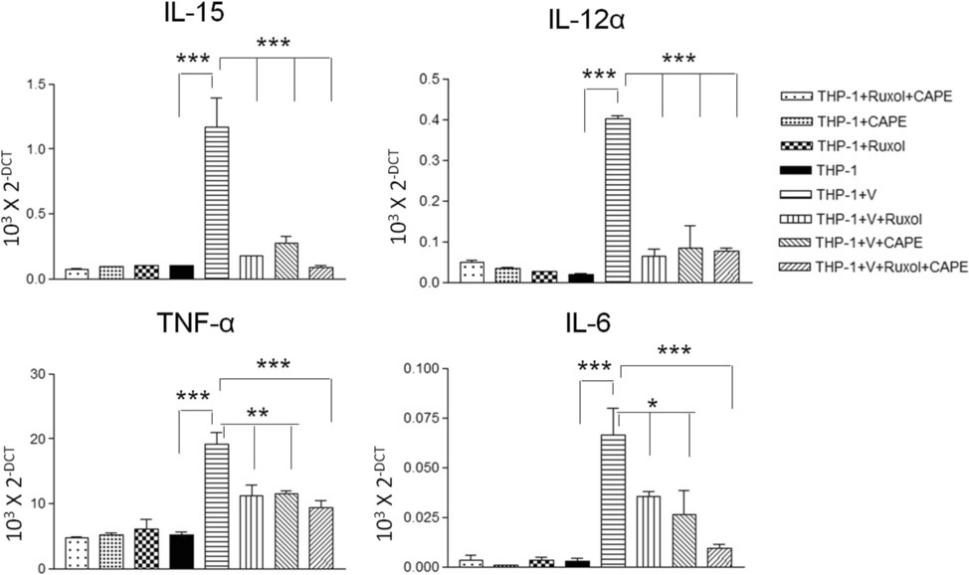
Ruxolitinib and CAPE block HD-Ad gene therapy mediated activation of macrophages. THP-1 cells were activated by 5 ng/ml PMA to prepare mature macrophages. Mature macrophages were harvested and cultured in presence and absence of ruxolitinib or CAPE, and in presence of both the inhibitors. As depicted in figure one cohort of these culture conditions were transduced with HD-Ad and other cohort was not transduced. After culturing them for 24 h total RNA was isolated and analysed by qRT- PCR to look for relative expression of different cytokines. Relative expression of cytokines from one experiment is depicted in the figure. All the data is normalised with GAPDH expression. The significance was calculated by one-way ANOVA with Tukey’s multiple comparison. *p < 0.05, **p < 0.01, ***p < 0.001

THP-1 cells were cultured in presence of Phorbol 12-myristate 13-acetate (PMA) for 48 h to differentiate them into macrophages. Differentiated THP-1 cells were harvested and cultured in presence of JAK inhibitor Ruxolitinib (1 μM) and NF-kB inhibiter CAPE (10 μM) for 24 h. Simultaneously, cohorts of these cells were also transduced with C4HSU HD-Ad vectors (5000 viral particles/cell). After 24 h of culturing them in presence of inhibitors, macrophages were harvested and total RNA was isolated and analyzed for expression of different cytokines by qRT- PCR analysis. Compared to untransduced macropahges, HD-Ad transduced cells showed significant increase in the expression of IL-15, IL-12α, TNF-α and IL-6 (p < 0.001) (Fig. 1). When macrophages were cultured in presence of ruxolitinib or CAPE, expression levels of IL-15, IL-12α, TNF-α and IL-6 decreased significantly compared to HD-Ad transduced cells without addition of inhibitors. When a combination of both ruxolitinib and CAPE were present, expression levels of IL-15decreased to basal levels as seen in untransduced cells (Fig. 1). Particularly expression of human IL-6 decreased very significantly, indicating additive effect of ruxolitinib and CAPE. Untransduced cells in the presence of inhibitors did not show any effect on cytokine gene expression. There was no secretion of IFN-γ by macrophages or significant difference in secretion of IL-1β, IL-8 and IL-18 between vector-transduced and -untransduced macrophages (results not shown). Human bronchial epithelial cells showed an increase in IL-1β, IL-8 secretion (Additional file 1: Figure S1) when they were transduced with HD-Ad vectors.

### JAK and NF-kB inhibitors block HD-Ad-mediated activation of NK cells in co-culture with macrophages

**Fig. 2:**
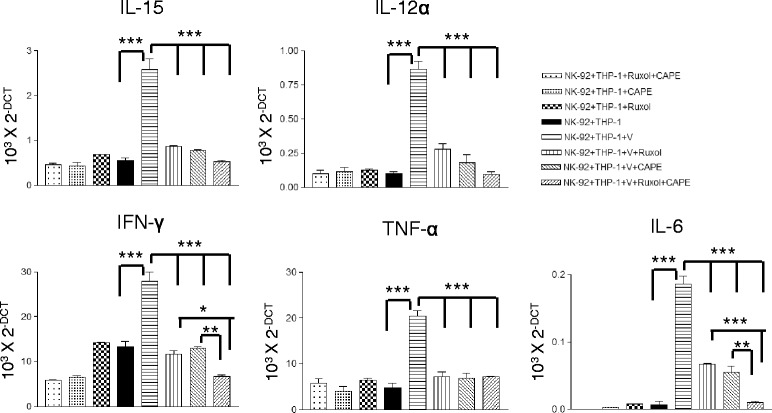
Ruxolitinib and CAPE block HD-Adv gene therapy mediated activation of NK cells by viral vector activated macrophages. NK-92 cells were deprived from IL-2 for 48 h and THP-1 cells were activated by 5 ng/ml PMA to prepare mature macrophages. Mature macrophages were harvested and cultured with NK-92 cells (1:1 ratio) in presence and absence of ruxolitinib or CAPE, and in presence of both the inhibitors. As depicted in figure two cohort of these culture conditions were transduced with HD-Adv and other cohort was not transduced. After culturing them for 24 h, total RNA was isolated and analysed by qRT- PCR to look for relative expression of different cytokines. Relative expression of cytokines from one experiment is depicted in the figure. All the data is normalised with GAPDH expression. The significance was calculated by one-way ANOVA with Tukey’s multiple comparison. *p < 0.05, **p < 0.01, ***p < 0.001

NK-92 cells were deprived from IL-2 for 48 h because NK −92 cells grown in presence of IL-2 are highly activated and are highly cytotoxic [31, 32]. It is well documented that IL-2 stimulation of NK cells induces increased granzyme B expression [33, 34]. Secretion of cytotoxic granules is the dominant mode of cytotoxicity associated with NK cells. Granzyme B plays an important role in targeted killing of susceptible cells in both human and mouse [35–37]. IL-2 deprived NK-92 cells are co-cultured with activated macrophages (PMA activated THP-1 cells) in 1:1 ratio. They are co-cultured in presence and absence of Ruxolitinib and CAPE inhibitors. Simultaneously, a cohort of co-cultured cells were also transduced with C4HSU HD-Ad vectors (5000 viral particles/cell). After 24 h culturing, total RNA was isolated from the co-cultured cells and analyzed for expression of different cytokines by qRT- PCR analysis. Upon HD-Ad vector transduction, there was a very significant increase in expression of IL-15,IFN-γ, IL-12α, TNF-α and IL-6 (p < 0.001) (Fig. 2). IFN-γ is the cytokine secreted by NK cells alone in our co-culture system, there was no expression in macrophages (data not shown). When vector transduced NK-92 cells alone were cultured in presence and absence of inhibitors, there was no significant difference in any cytokine secretion (results are not shown). There was a significant decrease in INF-γ expression by NK cells cultured in presence of JAK2 and NF-κB inhibitors. There was an additive effect on INF-γ secretion in co-cultured cells when both the inhibitors were used (Fig. 2). Apart from IFN-γ, other cytokines like IL-15, IL-12α, TNF-α and IL-6 levels were also significantly decreased in ruxolitinib and CAPE inhibitor added cells compared to no-inhibitor. Similar to macrophage mono culture results there was very significant additive effect of inhibitors on human IL-6 expression in combination inhibitors (Fig. 2). Unlike human bronchial epithelial cells, NK-92 and THP-1 co-culture did not show any significant difference in IL-1β, IL-8 and IL-18 secretion upon HD-Ad transduction (Additional file 1: Figure S1).

### NK cell mediated killing of HD-Ad transduced epithelial cells can be blocked by Ruxolitinib and CAPE

**Fig. 3:**
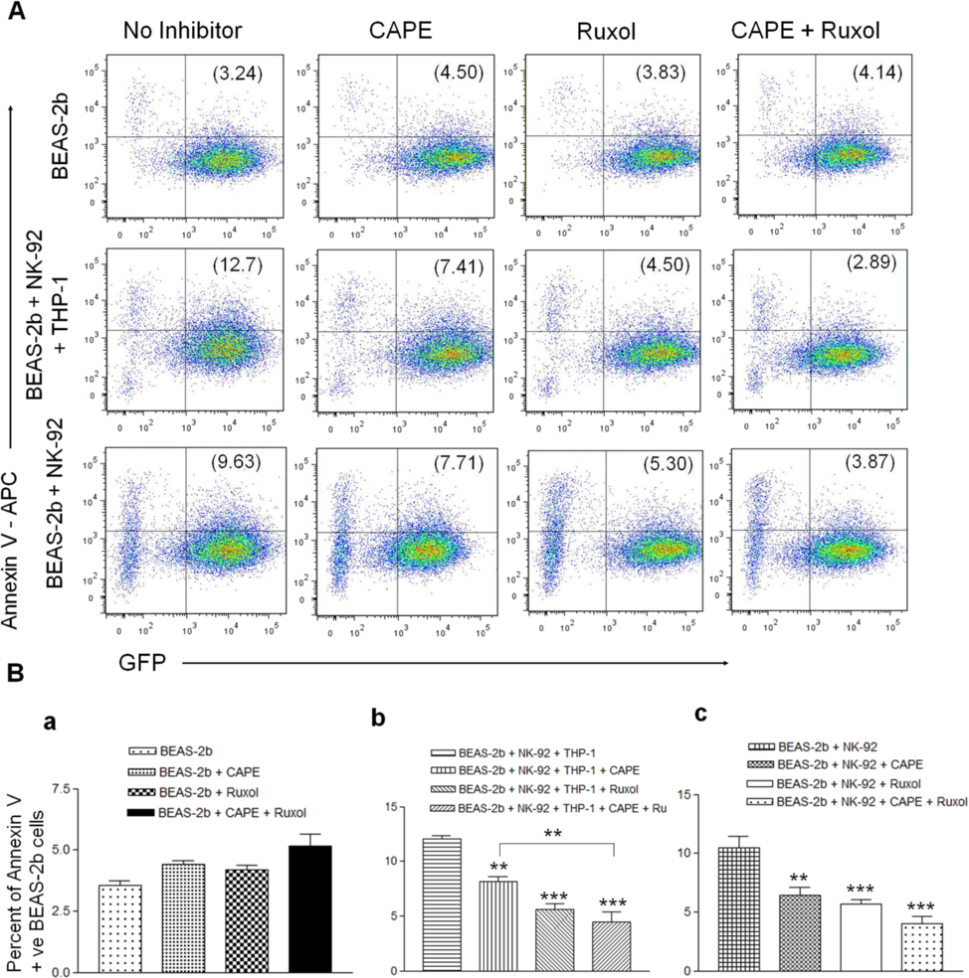
NK cell mediated apoptosis of HD-Ad gene transduced epithelial cells is blocked by Ruxolitinib and CAPE. NK-92 cells were deprived from IL-2 for 48 h and THP-1 cells were activated by 5 ng/ml PMA to prepare mature macrophages. Mature macrophages were harvested and co-cultured with NK-92 cells in presence of ruxolitinib or CAPE, and in presence and absence of both the inhibitors for 24 h. NK-92 cells alone were also cultured in presence of ruxolitinib or CAPE, and in presence and absence of both the inhibitors for 24 h. Both the cohorts of NK-92 and macrophages were transduced with C4HSU vector. Simultaneously, GFP-C4HSU transduced BEAS-2b cells were also cultured in presence of ruxolitinib or CAPE, and in presence and absence of both the inhibitors for 24 h. After culturing them for 24 h, NK-92 and macrophages were transferred to respective BEAS-2b cultures. After incubating those co-cultures for 5 h, BEAS-2b cells were harvested and stained for APC-Annexin V and 7AAD. Samples were run on flowcytometer and analysed by gating on GFP positive, gene transduced BEAS-2b cells. Percentages of apoptotic gene transduced BEAS-2b cells are depicted on upper right quadrant of panel (**a**). Percent apoptotic cells from each row are depicted as separate bar diagram in panel (**b**) to better depict significant differences. Results from one experiment is depicted in the figure. The significance was calculated by one-way ANOVA with Tukey’s multiple comparison. *p < 0.05, **p < 0.01, ***p < 0.001

**Fig. 4:**
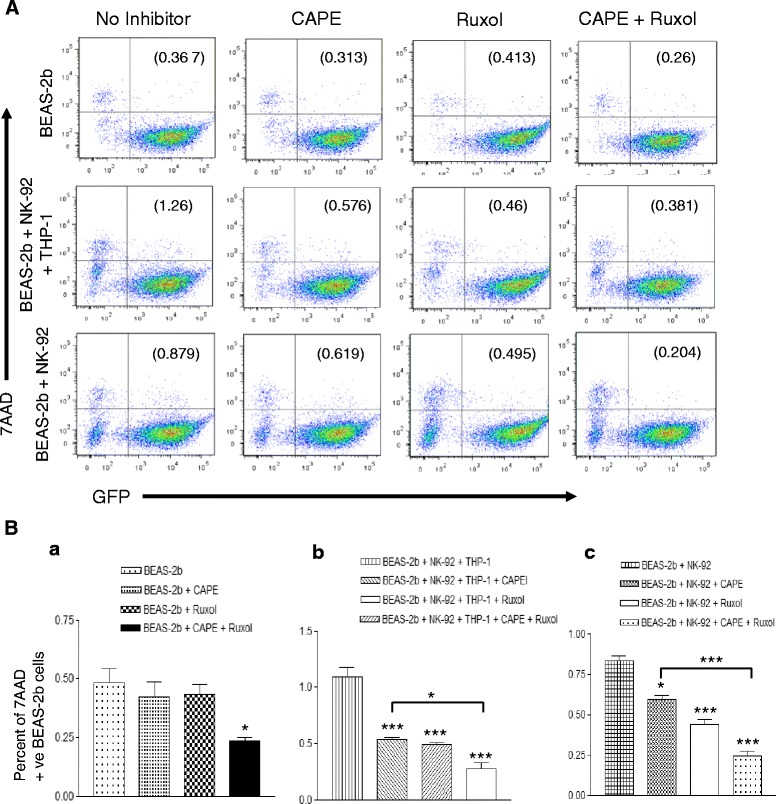
NK cell mediated killing of HD-Ad gene transduced epithelial cells is blocked by Ruxolitinib and CAPE. NK-92 cells were deprived from IL-2 for 48 h and THP-1 cells were activated by 5 ng/ml PMA to prepare mature macrophages. Mature macrophages were harvested and co-cultured with NK-92 cells in presence of ruxolitinib or CAPE, and in presence and absence of both the inhibitors for 24 h. NK-92 cells alone were also cultured in presence of ruxolitinib or CAPE, and in presence and absence of both the inhibitors for 24 h. Both the cohorts of NK-92 and macrophages were transduced with C4HSU vector. Simultaneously, GFP-C4HSU transduced BEAS-2b cells were also cultured in presence of ruxolitinib or CAPE, and in presence and absence of both the inhibitors for 24 h. After culturing them for 24 h, NK-92 and macrophages were transferred to respective BEAS-2b cultures. After incubating those co-cultures for 5 h, BEAS-2b cells were harvested and stained for APC-Annexin V and 7AAD. Samples were run on flowcytometer and analysed by gating on GFP positive, gene transduced BEAS-2b cells. Percentages of 7AAD positive dead gene transduced BEAS-2b cells are depicted on upper right quadrant of panel (**a**). Percent dead cells from each row are depicted as separate bar diagram in panel (**b**) to better depict significant differences. Results from one experiment is depicted in the figure. The significance was calculated by one-way ANOVA with Tukey’s multiple comparison. *p < 0.05, **p < 0.01, ***p < 0.001

Mature macrophages from activated THP-1 cells are co-cultured over night with IL-2 deprived NK-92 cells in presence of the empty HD-Ad vector (C4HSU). Macrophage activated NK-92 cells were then transferred to overnight GFP-C4HSU vector (5000 viral particles/cell) transduced BEAS-2b cells for cytotoxicity assay. Co-cultures were done in presence and absence of ruxolitinib and CAPE alone, and also in presence of both the inhibitors. These co-cultures were incubated for five hours. After incubation for cytotoxicity, BEAS-2b cells were harvested and stained for early apoptosis marker allophycocyanin (APC)-conjugated Annexin V (Fig. 3) and dead (permeable) cell marker, 7-Aminoactinomycin D (7AAD), (Fig. 4). Stained cells were analysed on flowcytometer by gating on GFP positive BEAS-2b cells.

In BEAS-2b monoculture incubated with inhibitors in combination or alone did not show significant difference in apoptosis of HD-Ad transduced cells (Fig. 3Ba). There was a slight reduction of dead cells in combination inhibitor treated BEAS-2b cells (Fig. 4Ba). NK-92 cells activated by mature THP-1 cells showed significant increase in apoptosis of HD-Ad transduced BEAS-2b cells, 12.7 % in co-cultured NK-92 cells compared to 3.24 % in absence of NK-92 cells (Fig. 3). In parallel, 7AAD stained dead cells were also significantly more in NK-92 and activated THP-1 co-cultured BEAS-2b cells, 1.26 % in co-cultured NK-92 cells compared to 0.367 % in absence of NK-92 cells (Fig. 4). NK-92 mediated killing was effectively blocked by Ruxolitinib and CAPE, with combination of both the inhibitor there was additive effect in inhibiting cytotoxicity by NK-92 cells. When HD-Ad trasnduced BEAS-2b cells are co-cultured with THP-1 and NK-92 cells there was significant apoptosis in no inhibitor cohort of cells (12.7 %) and it significantly decreased with CAPE (7.41 %), Ruxolitinib (4.50 %) and there was additive impact with both the inhibitors (2.89 %) (Fig. 3Bb). In concurrence with apoptotic cell number, GFP positive dead cells were also significantly more in no inhibitor cohort of cells (1.26 %) and their numbers decrease significantly in CAPE (0.576 %), ruxolitinib (0.46 %) and with both inhibitors (0.381) showing additive effect of combination inhibitors (Fig. 4Bb).

When we used HD-Ad transduced NK-92 cells alone in cytotoxicity assay, they also showed increased cytotoxicity towards HD-Ad transduced BEAS-2b cells. Apoptosis induced by NK-92 cells on BEAS-2b cells was significantly high in no inhibitor cohort of cells (9.63 %), apoptotic cell number significantly decreased with CAPE (7.71 %), ruxolitinib (5.30 %) and it was 3.87 % with both the inhibitors (Fig. 3Bc). There was additive effect on 7AAD positive killed BEAS-2b cells when we used combination of both the inhibitors. Killed cells were significantly high in no inhibitor cohort of cells (0.879 %) compared to CAPE (0.619 %), ruxolitinib (0.495 %) and with both the inhibitors it was 0.204 % (Fig. 4Bc).

## Discussions

Resident alveolar macrophages are abundant in lower respiratory tracts, acting as rapid first line of defense for invaders. Both macrophages and epithelial cells respond to viral infection by releasing soluble mediators, helping in the recruitment of innate and adaptive effector cells. In the process they recruit NK cells to the site of infection [38]. In an established mouse model of respiratory syncytial virus (RSV) disease, by depleting macrophages through clodronate liposome mediate depletion of macropahges. Macrophage depletion specifically affected NK cell recruitment, but not CD4 and CD8 T cells [38]. Up on viral infection macrophages have been shown to produce cytokines like IL-15, TNF-α and IL-12 [39–42]. Similarly, our HD-Ad vectors used for gene therapy did induce secretion of cytokines IL-15, IL-12, TNF-α and IL-6 when we transduced activated macrophages from THP-1 cells. Macrophages have also been shown to engulf viral vectors and destroy them through lysosomal degradation [43]; macrophage suppression may help in sustained gene expression in lung- gene therapy. In our study we have used small molecule blockers ruxolitinib and CAPE to block NF-κB and Jak-Stat pathways, respectively. Both ruxolitinib and CAPE have significant inhibition on cytokine secretion by macrophages. As both the inhibitors act on different pathways of activation, there was additive effect on inhibiting IL-6 and TNF-α secretion up on using combination of both the inhibitors. However, these findings are from an in vitro system, we need to futher confirm them in suitable *in vivo* system. Recently, ruxolitinib is shown to inhibit dendritic cell mediate immune response through decreased IL-12 production [30]. NF-κB inhibitor CAPE was shown to have inhibitory effect on the production of pro-inflammatory cytokines from LPS-stimulated macrophages by inhibiting cytokine expression [44].

Macrophages and dendritic cells (DCs) are shown to regulate the NK cell activity through production of type I interferons, IL-12 and IL-15 [17, 45–49]. Current understanding of NK cell development involves the association of antigen presenting cells (APCs) for NK cell activation, because NK cells are not matured in bone marrow, they come out immature and get activated by APCs [50]. When NK cells are co-cultured with activated macrophages, in presence of our HD-Ad, IFN-γ cytokine expression by NK cells increased significantly. IFN-γ is only secreted by NK cells in our co-culture, it did not show any significant variation when NK cells alone were transduced, indicating the suppressive effect of inhibitors CAPE and ruxolitinib. IFN-γ expression decreased significantly up on incubating them with these inhibitors. There was additive inhibition on INF-γ secretion when both the inhibitors were used. In Rag2−/−γc−/− mice transplanted with human hematopoietic stem cells, membrane bound IL-15 is shown to effectively stimulate NK cells in to cytotoxic effector cells [51]. Although soluble cytokine has some response membrane bound IL-15 has significant influence on NK differentiation [51, 52]. To evaluate effect of contact dependant stimulation in our model, we did co-culture NK cells and macrophages in transwells with NK cells at the top and activated THP-1 cells at the bottom well. Twenty-four hrs after incubation both the cells were harvested separately and looked for expression levels of cytokines (results are not shown). There was no difference in expression levels of cytokines, indicating that membrane bound IL-15 alone may not have any significant influence in our gene delivery system.

We have demonstrated that NK cells activated by cytokines produced by HD-Ad vector activated macrophages kill HD-Ad vector transduced bronchial epithelial cells. The apoptotic cell number and number of vector-transduced epithelial cells killed are significantly reduced by NF-κB inhibitor CAPE and also by JAK inhibitor ruxolitinib. Combination of these two inhibitors has an additive effect on inhibiting NK cell mediate killing of gene transduced cells. In our previous study we have seen extensive increase in the IFN-γ at 5 to 7 days, which is secreted by NK cells, in our HD-Ad mediated gene therapy of pig lungs. Various viral infection models have also shown that NK cell response peaks at one week [53, 54]. Transient inhibition of this NK cell response at its peak will enhance sustained gene expression.

## Conclusions

**Fig. 5:**
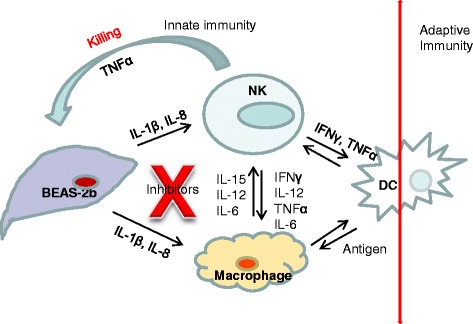
Model depicting the interaction of gene transduced bronchial epithelial cells with NK cells and macrophages. Cascade of events leading to killing of gene transduced bronchial epithelial cells can be broken by transiently inhibiting NK cell activation at its peak response in HD-Ad gene therapy. Apart from protecting gene transduced cells from NK cell mediated killing, this inhibition will also help in dampening the induction of adoptive immune response

Based on our study with human cell lines, combination of CAPE and ruxolitinib will help in protecting gene transduced air way epithelial cells to prolong transgene expression by curtailing NK cell mediated deletion of gene transduced cells. NK cells are also shown to play an important role in priming and regulation of adaptive immune responses [55]. As depicted in Fig. 5 effective inhibition of NK cell response at its peak will also inhibit HD-Ad capsid mediated adaptive immune response. Cutting the arm leading to adaptive immune response will help in the redelivery of HD-Ad vectors for gene therapy as dampened antigen presentation happens with suppressed macrophage and NK cell response [56, 57].

## Methods

### Cell lines and reagents

NK-92 is a human Natural Killer cell line derived from rapidly progressive non-Hodgkin's lymphoma patient's peripheral blood mononuclear cells [20]. NK-92 cells were cultured in minimum essential medium (MEM) alpha without ribonucleosides and deoxyribonucleosides (Lifetechnologies, Burlington, ON, Canada), with 2 mM L-glutamine (Lifetechnologies, Burlington, ON, Canada), 1.5 g/L sodium bicarbonate (Sigma Aldrich, Missouri, USA), 0.2 mM inositol (Sigma Aldrich, Missouri, USA), 0.1 mM 2-mercaptoethanol (Sigma Aldrich, Missouri, USA), 0.02 mM folic acid(Sigma Aldrich, Missouri, USA), 100 U/ml recombinant IL-2 (Peprotech, NJ, USA), 12.5 % horse serum (Wisent Inc. Quebec, Canada) and 12.5 % fetal bovine serum (FBS) (Lifetechnologies, Burlington, ON, Canada). THP-1, a monocyte cell line [58] was maintained in RPMI-1640 (Lifetechnologies, Burlington, ON, Canada) with 10 % FBS and 0.05 mM 2-mercaptoethanol. THP-1 cells were differentiated to mature macrophages in presence of 5 ng/ml Phorbol 12-myristate 13-acetate (PMA) for 48 h. PMA was obtained from Sigma Aldrich, Missouri, USA. BEAS-2b, a cell line established from normal human bronchial epithelial cells, was obtained from the American Type Culture Collection (ATCC). These cells were maintained in Dulbecco's Modified Eagle's Medium (DMEM) (Lifetechnologies, Burlington, ON, Canada) with 10 % FBS. Ruxolitinib, a Janus kinase inhibitor was obtained from LC laboratories Woburn, MA, USA. Caffeic acid phenethyl ester (CAPE) was obtained from Tocris bioscience, Bristol, United Kingdom.

### Helper dependant Adenoviral vector production

Design and production of the HD-Ad vectors expressing GFP and empty/pC4HSU vectors used in this study were described previously [12, 59]. Briefly a GFP gene expression cassette was cloned into the pC4HSU vector which was linearized with a restriction enzyme, Pme I, for viral production. HD-Ad vectors were produced and purified by CsCl gradient centrifugation.

### Quantitative Real time PCR

For mRNA expression analysis, total RNA was isolated form cells by using the Qiagen RNAeasy kit (Qiagen, Mississauga, Ontario, Canada) according to the manufacturer's instructions, followed by DNase digestion. One μg of total RNA obtained was reverse transcribed using random hexamers and SuperScript II reverse transcriptase ((Invitrogen, Carlsbad, CA, USA) following the manufacturer's protocol. Ten ng of cDNA were then used as template for real-time PCR using SYBR Green in ABI prism 7000 (Life Technologies Inc., Burlington, ON, Canada). The primers used for detecting different cytokine levels are as follows: hIL-15 Forward-CAGAAGCCAACTGGGTGAATG and Reverse- GGGTGAACATCACTTTCCGTATA, hIL-12α Forward-CGTCAGCAACATGCTCCAGAA and Reverse- GGCAACTCTCATTCTTGGTTAATTC, hIFN-γ F:orward-GGGTTCTCTTGGCTGTTACTG and Reverse- CTGTCACTCTCCTCTTTCCAATTC, hTNF-α Forward- GGTGCTTGTTCCTCAGCCTC and Reverse- GGTTCGAGAAGATGATCTGACTG, hIL6 Forward- GGATGCTTCCAATCTGGATTCAAT and Reverse- CTGCACAGCTCTGGCTTGTT.

### Flowcytometric apoptosis and cytotoxicity assay

NK-92 cells were cultured in complete NK-92 growth medium without IL-2 supplement for 48 h, to deprive them from IL-2. Simultaneously, THP-1 cells were differentiated into mature macrophages in presence of 5 ng/ml PMA for 48 h. After 48 h mature macrophages attached to culture plates were harvested by using Accutase (Sigma-Aldrich Canada Co. Oakville, Ontario Canada) and co-cultured with IL-2 deprived NK-92 cells in 1:1 concentration, in presence and absence of different inhibitors and C4HSU vector (5000 viral particles/cell) as depicted in figures, for one day. Mono cultures of IL-2 deprived NK-92 cells were also incubated with and without inhibitors, and C4HSU vector (5000 viral particles/cell) as depicted in figures, for one day. Simultaneously, one day after starting the IL-2 deprival for NK-92 and THP-1 differentiation to macrophages, BEAS-2b cells were plated on to 60 mm tissue culture dishes at 2X10^5^ cells per plate. Next day, media in those plates were replaced with or without GFP expressing vector (5000 viral particles/cell) as depicted in figure, in serum free RPMI-1640 for vector transduction. After two hours the media in all BEAS-2b plates were replaced with RPMI-1640 with 10 % FCS and cultured for one day. Later, co-cultured NK-92 and macrophages, and also mono cultured NK-92 were added to respective BEAS-2b plates as depicted in figures, in 5:1:1 (BEAS-2b: NK-92: THP-1) ratio. (Note: when we transfer co-cultured NK-92 and macrophages, though we mix and transfer those cells to BEAS-2b plates we are not transferring attached macrophages). Five hours after co-culturing these cells together, BEAS-2b cells were harvested using Trypsin EDTA (Lifetechnologies, Burlington, ON, Canada). Harvested cells were washed with PBS, and stained with Annexin V-APC (eBioscience Inc., San Diego, CA, USA) and 7AAD (eBioscience Inc., San Diego, CA, USA) for 30 min, in Annexin staining buffer (eBioscience Inc.) as per manufacturers protocol. After 30 min 300 μl of Annexin binding buffer was added and samples were analyzed on BD LSR-II flowcytometer (BD Biosciences, Mississauga, ON, Canada). GFP positive vector transduced BEAS-2b cells were gated and further analysed for apoptotic Annexin V and killed 7AAD positive population of GFP transduced BEAS-2b cells.

### Statistical analysis

All experiments were performed at least for 3 times, with representative experiments shown in figures. To test for statistical significance one-way ANOVA was done with Tukey's post hoc test, and Mann–Whitney test, using Prism 5 software.

Supplementary information is available at Cell and Biosciences website.

## Additional file

Additional file 1: Figure S1. HD-Adv transduced bronchial epithelial cells produce pro-inflamatory cytokines. One cohort of BEAS-2b cells were transduced with HD-Adv and other cohort was not transduced and cultured for 24 h. After culturing them for 24 h total RNA was isolated and analysed by qPCR to look for relative expression of different cytokines. Relative expression of cytokines from one experiment is depicted in the figure. The significance was calculated by Mann–Whitney *U* test.
