# Supplementary figures and images for: Transient blocking of NK cell function with small molecule inhibitors for helper dependant adenoviral vector-mediated gene delivery
Source: Cell Biosci. 2015 Jun 11;5:29. doi: 10.1186/s13578-015-0023-0 (PMC4470062; doi:10.1186/s13578-015-0023-0)

Supplementary figure 1

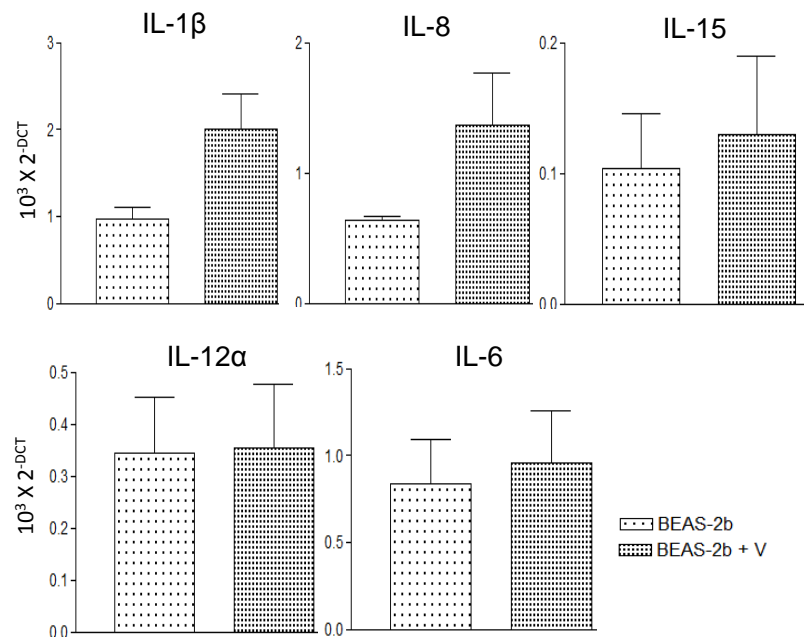

Supplement: Additional file 1: Figure S1. — HD-Adv transduced bronchial epithelial cells produce pro-inflamatory cytokines. One cohort of BEAS-2b cells were transduced with HD-Adv and other cohort was not transduced and cultured for 24 h. After culturing them for 24 h total RNA was isolated and analysed by qPCR to look for relative expression of different cytokines. Relative expression of cytokines from one experiment is depicted in the figure. The significance was calculated by Mann–Whitney U test. [file 13578_2015_23_MOESM1_ESM.pdf]
